# Pharmacological Activities of Ruthenium Complexes Related to Their NO Scavenging Properties

**DOI:** 10.3390/ijms17081254

**Published:** 2016-08-02

**Authors:** Anna Castellarin, Sonia Zorzet, Alberta Bergamo, Gianni Sava

**Affiliations:** 1 Callerio Foundation Onlus, via A. Fleming 22-31, 34127 Trieste, Italy; anna_fc@yahoo.it (A.C.); a.bergamo@callerio.org (A.B.); 2 Department of Life Sciences, University of Trieste, 34127 Trieste, Italy; zorzet@units.it

**Keywords:** ruthenium, angiogenesis, anticancer, nitric oxide, cell cultures

## Abstract

Angiogenesis is considered responsible for the growth of primary tumours and of their metastases. With the present study, the effects of three ruthenium compounds, potassiumchlorido (ethylendiamminotetraacetate)rutenate(III) (RuEDTA), sodium (bis-indazole)tetrachloro-ruthenate(III), Na[*trans*-RuCl_4_Ind_2_] (KP1339) and *trans*-imidazoledimethylsulphoxidetetrachloro-ruthenate (NAMI-A), are studied in vitro in models mimicking the angiogenic process. The ruthenium compounds reduced the production and the release of nitrosyls from either healthy macrophages and immortalized EA.hy926 endothelial cells. The effects of NAMI-A are qualitatively similar and sometimes quantitatively superior to those of RuEDTA and KP1339. NAMI-A reduces the production and release of nitric oxide (NO) by the EA.hy926 endothelial cells and correspondingly inhibits their invasive ability; it also strongly inhibits the angiogenesis in matrigel sponges implanted subcutaneously in healthy mice. Taken together, these data support the anti-angiogenic activity of the tested ruthenium compounds and they contribute to explain the selective activity of NAMI-A against solid tumour metastases, the tumour compartment on which angiogenesis is strongly involved. This anti-angiogenic effect may also contribute to the inhibition of the release of metastatic cells from the primary tumour. Investigations on the anti-angiogenic effects of NAMI-A at this level will increase knowledge of its pharmacological properties and it will give a further impulse to the development of this class of innovative metal-based drugs.

## 1. Introduction

The role of the angiogenic processes for cancer growth and dissemination has been long demonstrated and angiogenesis identified as a target for the pharmacological control of tumour malignancy [1,2,3,4]. A significant example of anti-angiogenic drug is Avastin (bevacizumab), a monoclonal antibody that changed the treatment paradigm of tumours such as colorectal cancer [5]. The pharmacological control of tumour angiogenesis, besides the use of specific monoclonal antibodies directed to vascular endothelial growth factor (VEGF) (see the above reported Avastin), can be controlled with tyrosine kinase inhibitors (TKIs, for example Sunitinib [6]) that block the signalling pathway that activates the angiogenic processes, and/or by the use of chemicals that interrupt the signalling between cells (for example Lenalidomide, the levo enantiomer of the old drug Thalidomide [7]). Independently of the type of drug being used, and of its mechanism of action, the main pharmacological effect expected is the arrest of the formation of new vessels, induced by the tumour, and correspondingly the block to the growth of the tumour because of the inhibited arrival of nutrients to the cells of the tumour mass.

Ruthenium-based drugs have also shown the ability to control the phenomena related to angiogenesis. In particular, imidazolium trans-imidazoledimethylsulphoxidetetrachloro-ruthenate (NAMI-A) inhibits the angiogenic process mimicked in the chick embryo chorioallantoic membrane (CAM) model [8] and in the pellets implanted in the rabbit cornea [9]. A more detailed study with the endothelial cell line ECV304 has clarified how NAMI-A was able to inhibit the growth of these cells through the inhibition of the receptor activated signal-extracellular regulated kinase (RAS-ERK) pathway at concentrations compatible with those reached in vivo in the metastatic sites [10,11,12]. The mechanism by which NAMI-A can control tumour angiogenesis has not been elucidated yet and support to this activity only comes from the observation that it is able to bind nitric oxide (NO), then reducing the activity of this gaseous transmitter that cancer cells use to modulate the angiogenesis on the endothelial cells [13,14,15].

The aim of the present study was therefore that of examining the pharmacological actions of NAMI-A, compared to those of two other ruthenium-based compounds (KP1339 and RuEDTA), in relation to their capacity to scavenge the nitric oxide. It is important to remember that the scavenging activity of NO of these compounds has already been studied through fourier transform infrared spectroscopy (FT-IR) and ^1^H-nuclear magnetic resonance (^1^H-NMR) spectroscopy techniques [9]. Here, the study will focus on the use of two in vitro cell models, the peritoneal murine macrophages (a cell population present in the tumour masses and to which the capacity to produce and release large quantities of NO is attributed [16]) and the human endothelial-like immortalised cell line EA.hy926 [17], and on the use of rat aorta rings cultured in vitro. Matrigel™ pellets implanted subcutaneously in mice will add further data from in vivo studies.

## 2. Results

### 2.1. Murine Peritoneal Macrophage Model

#### Effects on Nitric Oxide (NO) Production

**Figure 1:**
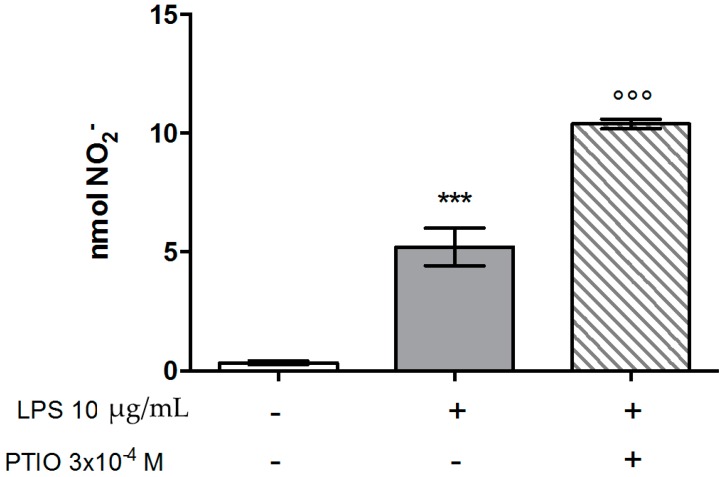
Nitric oxide (NO) release in the extracellular medium by macrophages. Murine peritoneal macrophages were treated for 24 h with 10 μg/mL lipopolysaccharide (LPS) and with 3 × 10^−4^ M 2-phenyl-4,4,5,5-tetramethylimidazoline-1-oxyl 3-oxide (PTIO), then NO release in the extracellular medium was measured by the Griess test. Data are the mean ± standard error medium (S.E.M.) of five samples per group. Unpaired *t*-test: *** *p* < 0.001 vs. no LPS no PTIO; °°° *p* < 0.001 vs. LPS.

**Figure 2:**
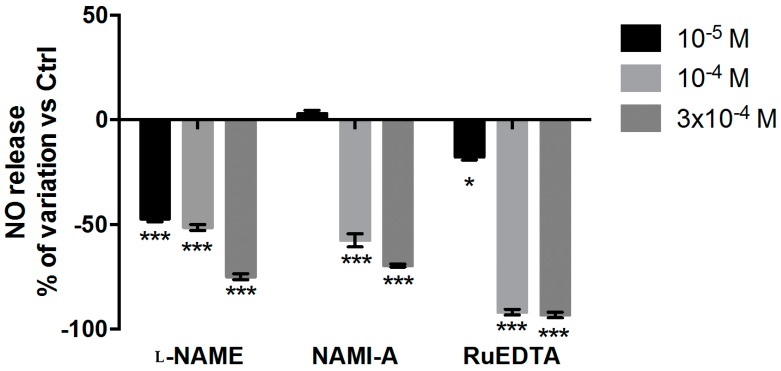
Effects of ruthenium compounds on the NO production by macrophages. Murine peritoneal macrophages were treated for 24 h with 10 μg/mL LPS and contemporary with 10^−5^, 10^−4^, and 3 × 10^−4^ M of *N*ω-nitro-l-arginine methyl ester hydrochloride (l-NAME), imidazolium *trans*-imidazoledimethylsulfoxidetetrachloro-ruthenate (NAMI-A), and potassiumchlorido(ethylendiamminotetraacetate)rutenate(III) (RuEDTA). NO release in the extracellular medium was measured by the Griess test. Data are expressed as per cent of variation vs. the controls and are the mean ± S.E.M. of five samples per group. ANOVA and Tukey–Kramer: * *p* < 0.05, *** *p* < 0.001 vs. Control.

**Figure 3:**
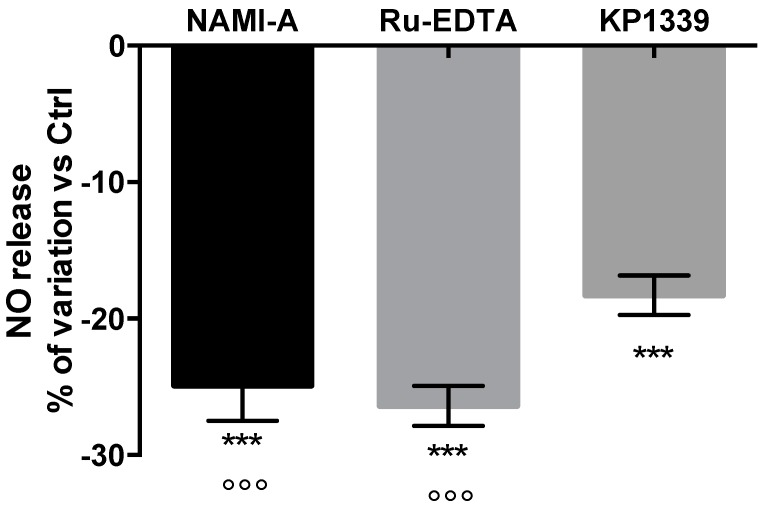
Effects of the pre-treatment with the ruthenium compounds on the NO production by macrophages. Murine peritoneal macrophages were treated with 10^−4^ M NAMI-A, RuEDTA, and sodium (bis-indazole)tetrachloro-ruthenate(III), Na[*trans*-RuCl_4_Ind_2_] (KP1339), then washed and further treated for 24 h with 10 μg/mL LPS. NO release in the extracellular medium was measured by the Griess test. Data are expressed as per cent of variation vs. the controls and are the mean ± S.E.M. of five samples per group. ANOVA and Tukey–Kramer: *** *p* < 0.001 vs. Control; °°° *p* < 0.001 vs. KP1339.

Upon treatment with lipopolysaccharide (LPS), which is known to stimulate the inducible form of nitric oxide synthase (NOS), murine peritoneal macrophages are strongly activated to produce nitric oxide (NO) (Figure 1). The contemporary treatment with 2-phenyl-4,4,5,5-tetramethylimidazoline-1-oxyl 3-oxide (PTIO) (used as the NO scavenger) significantly increased the extracellular NO_2_^−^ concentration as detected by the Griess test. This effect is consequent to the mechanism by which PTIO exerts its NO scavenging activity; PTIO oxidises the excess of NO released in the extracellular medium, transforming it into NO_2_^−^, which is then detected by the Griess test as an increase of NO_2_^−^ concentration (Figure 1). The treatment of murine peritoneal macrophages with 10^−4^ and 3 × 10^−4^ M NAMI-A significantly reduced the release of NO in the extracellular medium, that had been induced by the contemporary stimulation with LPS (Figure 2). The effect is comparable to that of equal concentrations of *N*ω-nitro-l-arginine methyl ester hydrochloride (l-NAME), the well-known inhibitor of NOS. The treatment with potassiumchlorido (ethylendiamminotetraacetate)rutenate(III) (RuEDTA) is even more effective, showing a reduction of NO release of about 90% in comparison to untreated controls. The ruthenium compounds maintained their ability to lower the NO release also when the cells were treated before being activated with LPS (Figure 3). In this case, the reduction of NO release was quantitatively less relevant than that measured in the contemporary treatment reported in Figure 2, and it was similar for NAMI-A and RuEDTA (reduction of approximately 25%). The pre-treatment with sodium (bis-indazole)tetrachloro-ruthenate(III), Na[*trans*-RuCl_4_Ind_2_] (KP1339) was somewhat less effective.

### 2.2. EA.Hy926 Endothelial Cell Line Model

#### 2.2.1. Cytotoxicity Test

**Figure 4:**
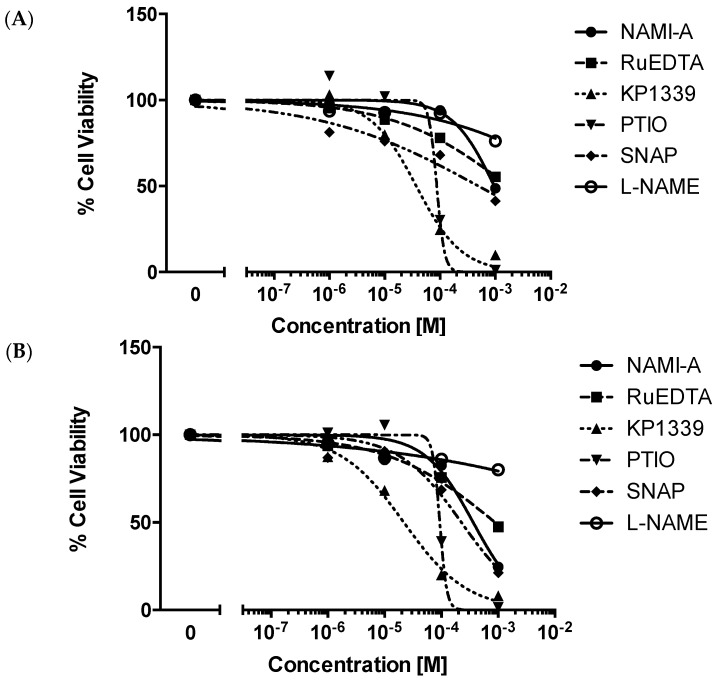
EA.hy926 cell viability after treatment with NAMI-A, RuEDTA, KP1339, PTIO, SNAP, and l-NAME. EA.hy926 cells were treated with compounds 10^−6^–10^−3^ M for: 48 h (**A**); or 72 h (**B**). Cell viability was measured with the 3-(4,5-dimethylthiazol-2-yl)-2,5-diphenyltetrazolium bromide (MTT) test.

Before examining the activity of the ruthenium compounds on the NO production of the EA.hy926 endothelial cell line, we studied their effects on the cell viability after cell treatment for 48 or 72 h (Figure 4A,B, respectively). The same test was done with the NO scavenger PTIO, with the NOS inhibitor l-NAME, and with the NO donor *S*-nitroso-*N*-acetyl-dl-penicillamine (SNAP), using the same experimental conditions. Among the ruthenium compounds tested, KP1339 was the most cytotoxic, with IC_50_ values in the 10^−5^ M range after either 48 or 72 h of cell exposure (Table 1). NAMI-A and RuEDTA showed a quite similar activity on the endothelial cell viability being able to significantly reduce it only after treatment at the highest concentration tested (10^−3^ M). The NOS inhibitor, l-NAME, was virtually devoid of any cytotoxicity up to the maximum concentration used, i.e., 10^−3^ M. SNAP and PTIO significantly affected cell viability after treatments at the two highest concentrations tested (10^−4^ and 10^−3^ M).

#### 2.2.2. Cell Invasion Test

**Figure 5:**
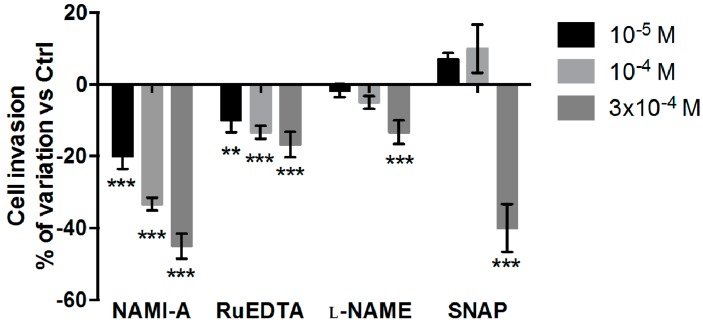
Effects of NAMI-A, RuEDTA, l-NAME, and SNAP on the invasive ability of EA.hy926 cells. EA.hy926 cells were treated with compounds at 10^−5^, 10^−4^, and 3 × 10^−4^ M for 24 h before seeding them on Transwell^®^ filters. Cell invasion was detected after further 24 h at 37 °C 5% CO_2_. Data are expressed as per cent of variation vs. the controls and are the mean ± S.E.M. of three samples per group. ANOVA and Tukey–Kramer: ** *p* < 0.01, *** *p* < 0.001 vs. Control.

NAMI-A significantly reduced, in a concentration-dependent manner, the invasive ability of the EA.hy926 endothelial cells (Figure 5). RuEDTA was also effective, although to a lesser extent (−17% at 3 × 10^−4^ M compared to −45% of NAMI-A at equimolar concentrations), and its activity was similar to that of the NOS inhibitor l-NAME. The NO donor SNAP significantly decreased the invasion of the endothelial cells, although this effect seemed mostly related to its cytotoxicity, consequent to the consistent release of NO that it induced at the highest concentration tested.

#### 2.2.3. NO Production and Release

**Figure 6:**
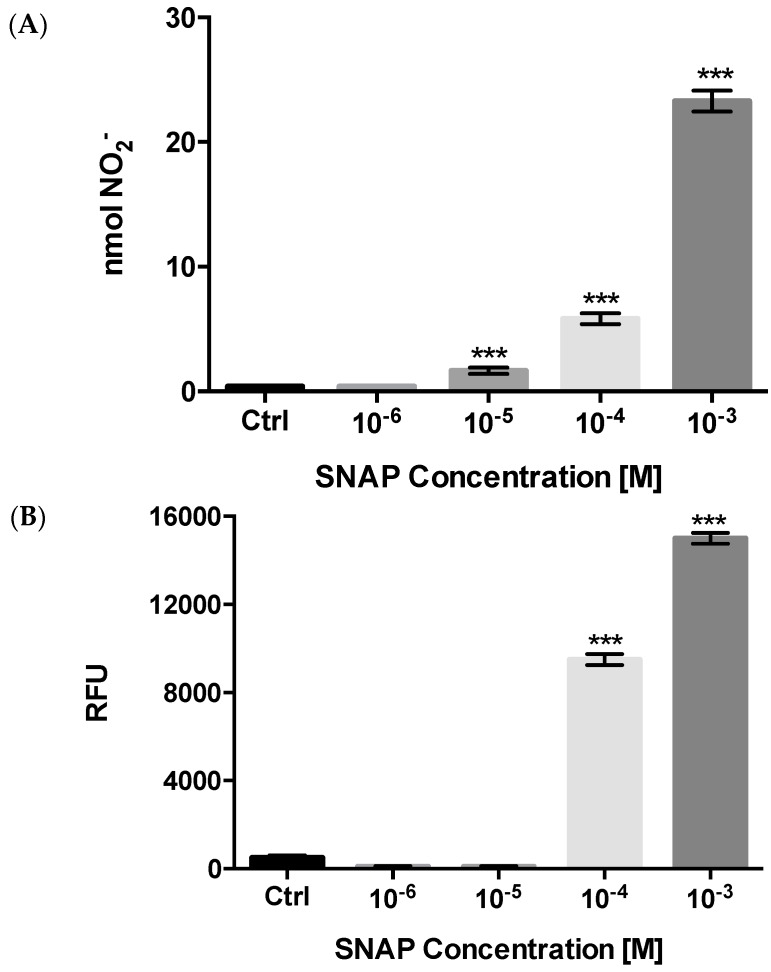
NO concentration at extracellular and intracellular level in EA.hy926 cells treated with SNAP. EA.hy926 cells were treated with SNAP 10^−6^–10^−3^ M for 2 h. Then, NO was quantified in the extracellular medium by Griess test (**A**) or at intracellular level by the fluorescent dye DAF-2 DA and expressed as Relative Fluorescence Units (RFU); (**B**) Data are the mean ± S.E.M. of five samples per group. ANOVA and Tukey–Kramer: *** *p* < 0.001 vs. Control.

The basal release of NO by EA.hy926 cells is rather low; therefore an experimental condition of high level of exogenous NO was generated using the NO donor compound SNAP. To maximally limit the drawbacks of the production of NO at non-physiological levels, the time exposure of cells to SNAP was secured at 2 h. With these experimental conditions, SNAP significantly increased the NO levels starting from 10^−5^ and 10^−4^ M, respectively, in the extracellular medium (Figure 6A) and inside the treated cells (Figure 6B). From this preliminary experiment a concentration of 10^−4^ M SNAP was judged to be the most appropriate for the subsequent experiments.

**Figure 7:**
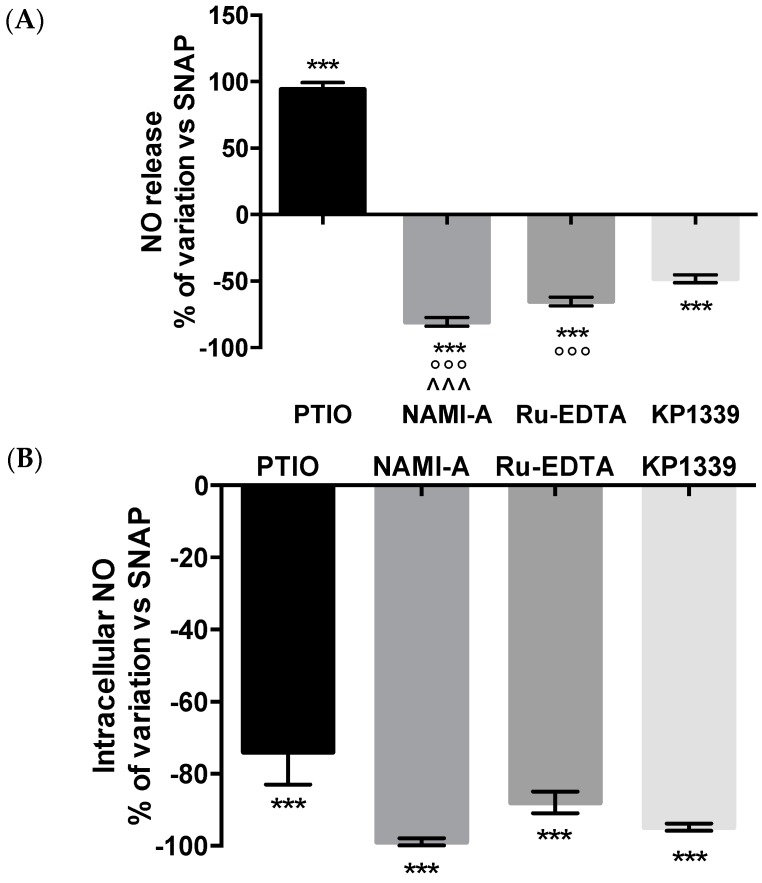
Effects of the contemporary treatment with NAMI-A, Ru-EDTA, KP1339, and PTIO on SNAP induced NO production/release by EA.hy926 cells. EA.hy926 cells were treated with 10^−4^ M SNAP ± an equimolar concentration of compounds for 2 h. Then, NO was measured in the extracellular medium by Griess test (**A**) or at intracellular level by the fluorescent dye DAF-2 DA; (**B**) Data are expressed as per cent of variation vs. SNAP treated cells and are the mean ± S.E.M. of five samples per group. ANOVA and Tukey–Kramer: *** *p* < 0.001 vs. Control, °°° *p* < 0.001 vs. KP1339; ^^^ *p* < 0.001 vs. Ru-EDTA.

**Figure 8:**
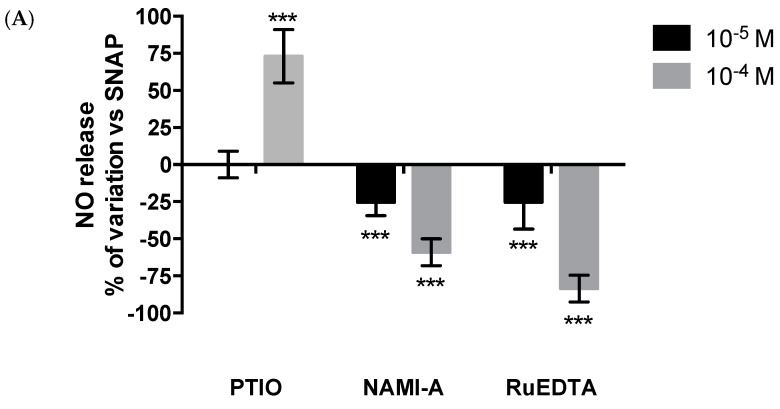

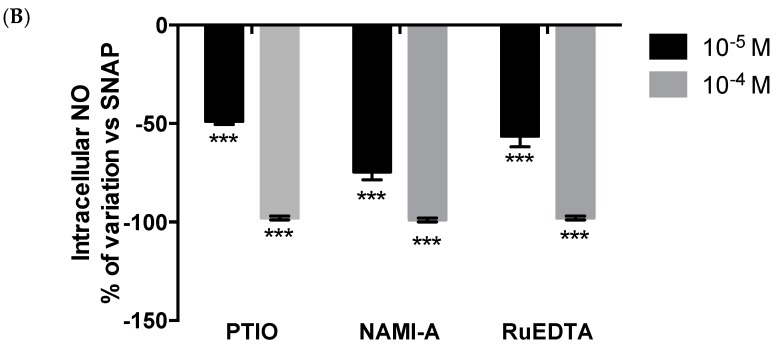
Concentration-dependent effects of the contemporary treatment with NAMI-A, Ru-EDTA, and PTIO on SNAP induced NO production/release by EA.hy926 cells. EA.hy926 cells were treated with 3 × 10^−4^ M SNAP ± 10^−5^ and 10^−4^ M concentration of compounds for 2 h. Then, NO was measured in the extracellular medium by Griess test (**A**) or at intracellular level by the fluorescent dye DAF-2 DA; (**B**) Data are expressed as per cent of variation vs. SNAP treated cells and are the mean ± S.E.M. of five samples per group. ANOVA and Tukey–Kramer: *** *p* < 0.001 vs. Control.

The treatment of the EA.hy926 endothelial cells with 10^−4^ M NAMI-A, RuEDTA and KP1339 for 2 h significantly counteracted the NO increase induced by the contemporary treatment with an equimolar concentration of SNAP in either the extracellular medium and intracellularly (Figure 7A,B). At the intracellular level, the NO scavenging ability of the ruthenium compounds was comparable, and even more pronounced than that of the standard NO scavenger PTIO. The increase of NO release in the extracellular medium upon PTIO treatment (Figure 7A) was the consequence of its mechanism of action, as already reported above. The NO scavenging ability of the ruthenium compounds during the contemporary exposure to the NO donor SNAP was further investigated to verify the relationship between the effect and the concentration tested (Figure 8A,B); NAMI-A and RuEDTA showed a concentration-dependent reduction of NO at either the extracellular and intracellular levels.

**Figure 9:**
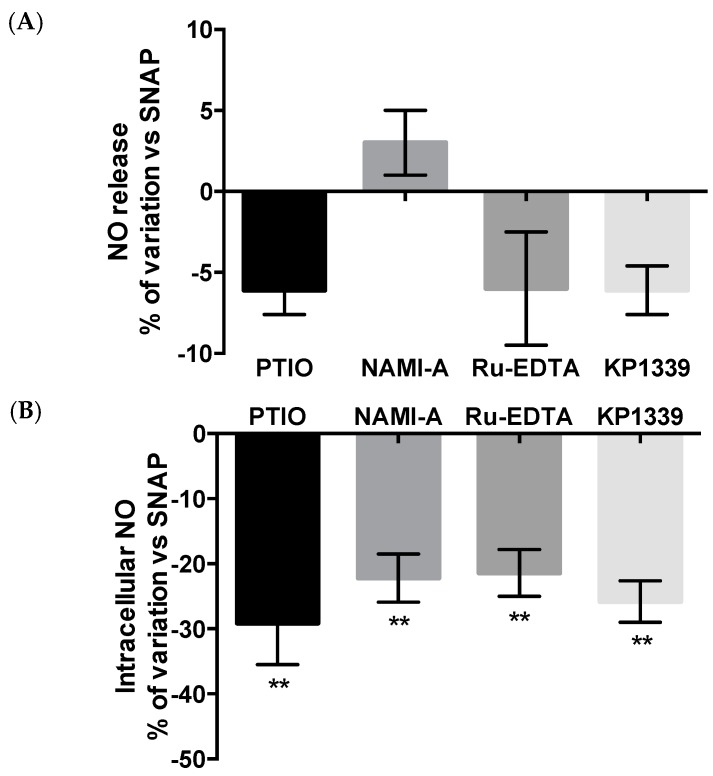
Effects of the pre-treatment with NAMI-A, Ru-EDTA, KP1339, and PTIO on SNAP induced NO production/release by EA.hy926 cells. EA.hy926 cells were pre-treated with 10^−4^ M NAMI-A, Ru-EDTA, KP1339, and PTIO for 2 h, then, after washing, with an equimolar concentration of SNAP for further 2 h. NO was measured in the extracellular medium by Griess test (**A**) or at intracellular level by the fluorescent dye DAF-2 DA; (**B**) Data are expressed as per cent of variation vs. SNAP treated cells and are the mean ± S.E.M. of five samples per group. ANOVA and Tukey–Kramer: ** *p* < 0.01 vs. Control.

The treatment with NAMI-A, RuEDTA, and KP1339, as well as with the positive control PTIO, for 2 h before the cell exposure to the NO donor SNAP, maintained their ability to reduce the NO levels inside the cells (Figure 9B). The effect was similar for the three ruthenium compounds, although quantitatively attenuated, in comparison to that observed with the contemporary treatment (approximately −25% vs. −90%; compare Figure 9B and Figure 7B). As expected, the pre-treatment with the ruthenium compounds, as well with PTIO, was completely ineffective to modulate the NO levels in the extra-cellular medium (Figure 9A).

**Figure 10:**
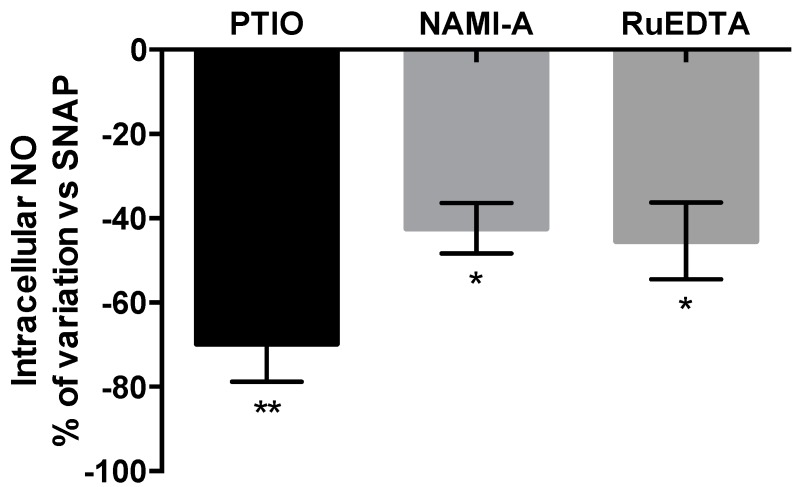
Effects of the post-treatment with NAMI-A, Ru-EDTA, and PTIO on SNAP induced NO production/release by EA.hy926 cells. EA.hy926 cells were pre-treated with 10^−4^ M SNAP for 2 h and then, after washing, with NAMI-A, Ru-EDTA, and PTIO for further 2 h. Then, NO was measured at intracellular level by the fluorescent dye DAF-2 DA. Data are expressed as per cent of variation vs. SNAP treated cells and are the mean ± S.E.M. of five samples per group. ANOVA and Tukey–Kramer: * *p* < 0.05, ** *p* < 0.01 vs. Control.

The NO scavenging ability of NAMI-A and RuEDTA at the intracellular level was measurable and it was relevant also when the treatment with the NO donor SNAP preceded the cell exposure to the ruthenium compounds (Figure 10). Once again the activity of the ruthenium compounds was quantitatively comparable (approximately −45% independently of the compound being tested). As expected, the detection of NO in the extracellular medium of control cells was under the threshold level (data not shown).

### 2.3. Anti-Angiogenic Activity of NAMI-A in the Model of Matrigel™ Pellets

**Figure 11:**
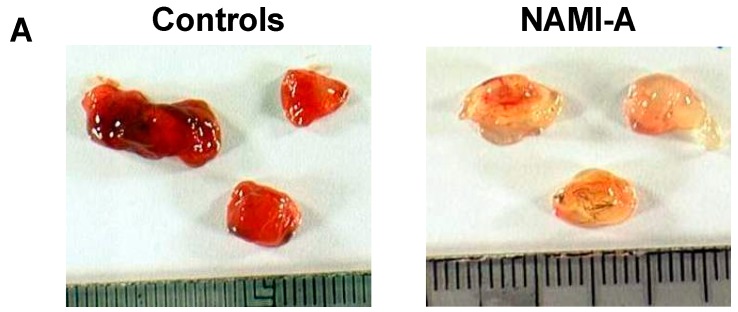

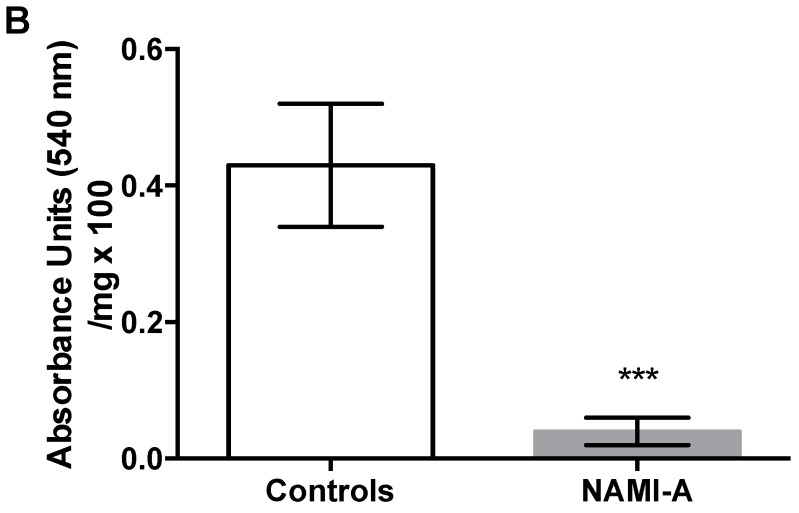
Anti-angiogenic activity of NAMI-A in the model of Matrigel™ pellets. The Matrigel™ pellets containing vascular endothelial growth factor (VEGF), heparin, TNF-α and phosphate buffered saline (PBS) (controls), or 2 × 10^−4^ M NAMI-A (treated) were implanted subcutaneously (s.c.) in C57/BL 6N mice. After four days, the pellets were explanted, photographed (**A**) and the haemoglobin content measured with the method of Drabkin; (**B**) Data are the mean ± S.E.M. of three samples. Unpaired *t*-test: *** *p* < 0.001 vs. Controls.

The morphological analysis of control Matrigel™ pellets showed a remarkable angiogenic activity, characterized by a diffuse network of blood vessels and an overt red colour (Figure 11A, left). In contrast, the pellets containing NAMI-A presented a pale coloration, indicative of a lesser presence of vessels (Figure 11A, right). This observation was confirmed by the quantitative analysis of the haemoglobin content in the pellets that was remarkably lower (−90%) in the treated group (Figure 11B).

## 3. Discussion

A significant impulse to the study and development of anticancer complexes of ruthenium has been given by the pioneering works with the so-called “symmetrical bis-heterocycles ruthenium(III)” [18] and with the dimethylsulphoxide-containing ruthenium(II) and later ruthenium(III) compounds [19,20]. If the initial idea was to mimic cisplatin in either potency or mechanism of action, suddenly the “ruthenium-sulphoxides” showed a low level of cell cytotoxicity in vitro [21] and a relatively modest activity against the primary sites of growth of syngenic mouse tumours in vivo [22]. On the contrary, these metal-based compounds exhibited an innovative capacity to control the development of secondary tumours (metastases) with either mouse models of solid tumours [23,24] and leukaemias [25]. The progress of knowledge on the contribution of angiogenesis to the development of tumour metastases prompted us to verify whether the anti-metastatic properties of NAMI-A (the most studied ruthenium-based complex on mouse models of tumour metastases [22,23,24,25,26,27]) could be ascribed to the control of the angiogenic process. These studies showed NAMI-A capable of inhibiting angiogenesis in all the test systems employed, namely the CAM model [8], the rabbit cornea model [9] and the Matrigel™ sponges implanted subcutaneously in the mouse of the present study.

NO is a key factor that stimulates the migration of endothelial cells during the angiogenesis process [13,28] and the interaction of ruthenium complexes with the nitrosyl ligand has been widely investigated [29,30,31]. All the ruthenium complexes tested in the present study have proven to control the production and release of NO by cells involved in the angiogenic processes facilitating the growth of tumours such as the macrophages and the endothelial cells (these latter here represented by the immortalized EA.hy926 cell line). It could be speculatively said that the anti-angiogenic activity of the complexes of the present study is due to their capacity to bind this small gaseous molecule as already demonstrated with the infrared spectroscopy [9]. In the case of the ruthenium complex NAMI-A, support to the role of NO scavenging for its innovative anti-tumour properties is given by the study of Das and Mondal [32] who have stressed the capacity of the adduct of NAMI-A with serum albumin to efficiently bind the nitrosyls, then immediately to undergo reduction to the more reactive ruthenium(II), a phenomenon that in the mind of these authors would explain the anti-metastatic properties of this drug. However, if the scavenging of extracellular NO can be easily understood, it is questionable that the reduction of the intracellular NO may depend on the same mechanism, given the demonstrated NAMI-A inability to penetrate into the cells [33].

Although capable of scavenging NO similarly to NAMI-A, RuEDTA and KP1339 do not share the same ability of NAMI-A to inhibit the endothelial cell invasion, suggesting that mechanisms others than NO scavenging are likely responsible of this activity, such as the reduced production/activation of MMPs caused by NAMI-A but not by the other ruthenium compounds ([8]; Callerio Foundation Onlus, data on file) It descends that the control of the production/release of NO is not sufficient to claim for anti-angiogenic properties for a given compound that correspondingly cannot be charged with capacities of controlling all the tumour cell activities related to angiogenesis. The anti-angiogenic effects of NAMI-A, here surrogated by the in vitro reduced invasive ability of EA.hy926 endothelial cells, are confirmed by the in vivo Matrigel™ pellets experiment, and further supported by a parallel study with aorta rings cultivated in vitro and exposed to increasing concentrations of the ruthenium-based drug. The results of this study can be summarized as follows: rat aorta rings obtained from healthy adult Wistar male rats, cultivated in vitro at 37 °C in Petri dishes over a Matrigel™ layer, allow the endothelial cells to grow forming elongated chains of cells invading the Matrigel™ structure. The addition of NAMI-A significantly reduces endothelial cell outgrowth from aorta rings in a dose-dependent manner and up to a complete suppression of the phenomenon at 3 × 10^−4^ M. The model was checked with SNAP (positive control that induces a pronounced increase of endothelial cell growth around the aorta ring) and with l-NAME (significant inhibition of the growth of endothelial cells around the aorta rings) (Callerio Foundation Onlus, data on file). This result confirms what already reported on the anti-angiogenic activity of NAMI-A adding a further model of angiogenesis in which the phenomenon is perturbed by concentrations of NAMI-A attainable with those obtained in vivo in the organs where tumour metastases are formed and grow [12].

The use and the results obtained with the reference standards PTIO, l-NAME and SNAP confirm the adequacy of the experimental protocols used to test the activity of NAMI-A and of the two other ruthenium complexes on the modulation of NO production. As expected, l-NAME reduces and SNAP increases the eNOS expression in the tested cells, as resulting from a series of Western blot analyses (Callerio Foundation Onlus, data on file). Therefore, the effects of NAMI-A and RuEDTA (marked reduction of eNOS expression) have pharmacological consistency. Similarly, the effect of l-NAME on the aorta rings cultivated in vitro (inhibition of the growth of endothelial structures), and those of SNAP (consistent promotion of endothelial growth), strengthen the meaning of the activity observed with NAMI-A on the same model and they provide for this drug evidence of the link between inhibition of eNOS, reduction of NO levels and inhibition of the angiogenesis.

In conclusion, even examining the angiogenesis, NAMI-A proves to have unique qualities not shared with other ruthenium complexes, and is also very similar to the compound KP1339. The anti-angiogenic properties of NAMI-A are attributable in part to its capacity to remove the extracellular NO, even when the drug is bound to serum albumin (the greater amount of the drug in the blood after intravenous injection [34]), and in part to its ability to modify the activity of transcription factors responsible for the production of NO, as shown in cells cultured in vitro for 1 h in the presence of anti-metastatic concentrations of the drug [35]. Although these data are not meant to attribute the anti-metastatic properties of NAMI-A solely to one’s ability to exert anti-angiogenic activity, they unequivocally show the strong capacity of this drug to counteract the tumour cell angiogenesis. It is less clear why this property contributes to the reduction of tumour metastases, while it apparently does not apply on the primary site of growth of solid tumours, on which the angiogenesis is similarly important, and on which the effects of NAMI-A have always been quite moderate if not completely null. If it is not excluded that the anti-angiogenic effect of NAMI-A may contribute to the inhibition of the release of metastatic cells from the primary tumour, it is almost surprising that the reduction of angiogenesis at this level does not induce also suffering of tumour cells at this site. Clarifying this aspect will increase the knowledge on the pharmacological properties of NAMI-A and will give a further impulse to the development of this class of innovative metal-based drugs.

## 4. Materials and Methods

### 4.1. Compounds and Chemicals

NAMI-A, imidazolium *trans*-imidazoledimethylsulfoxidetetrachlororuthenate(III), ImH [*trans*-RuCl_4_(DMSO)(Im)], was prepared according to the published procedure [36]. RuEDTA, potassiumchlorido(ethylendiamminotetraacetate)rutenate(III), K[Ru(HEDTA)Cl], was prepared according to the published procedure [37] and kindly provided by the group of Luigi Messori, University of Florence (Florence, Italy). KP1339, sodium (bis-indazole)tetrachloro-ruthenate(III), Na[*trans*-RuCl_4_Ind_2_], was kindly provided by Bernhard Keppler, University of Vienna (Vienna, Austria).

2-Phenyl-4,4,5,5-tetramethylimidazoline-1-oxyl 3-oxide (PTIO), *S*-nitroso-*N*-acetyl-dl-penicillamine (SNAP), *N*ω-nitro-l-arginine methyl ester hydrochloride (l-NAME) and all other reagents were purchased from Sigma-Aldrich (St. Louis, MO, USA), unless otherwise indicated.

**Scheme 1:**
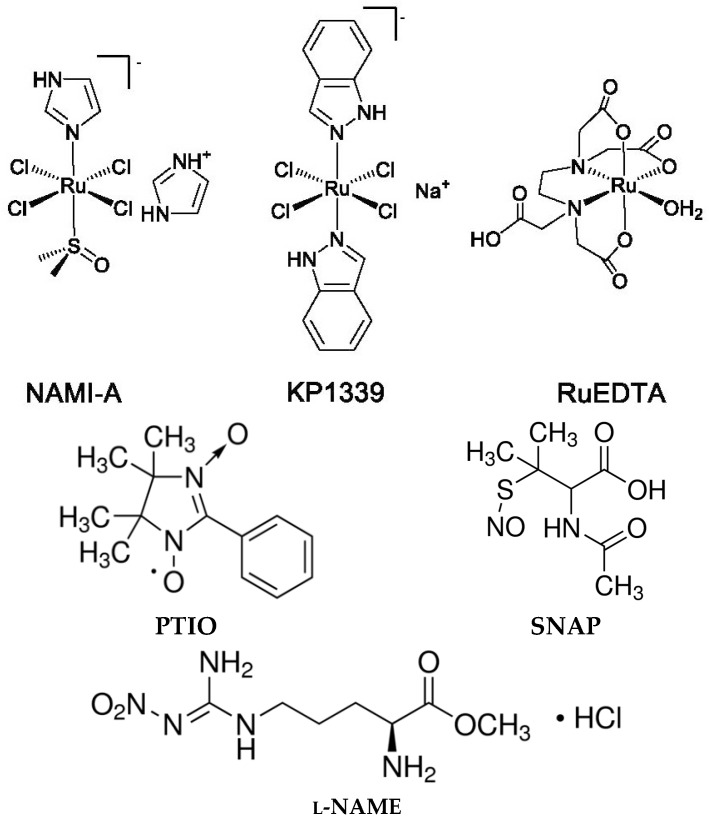
Schematic representation of the chemical structure of *trans*-imidazoledimethylsulphoxidetetrachloro-ruthenate (NAMI-A), sodium (bis-indazole)tetrachloro-ruthenate(III), Na[*trans*-RuCl_4_Ind_2_] (KP1339), potassiumchlorido(ethylendiamminotetraacetate)rutenate(III) (RuEDTA), and of 2-phenyl-4,4,5,5-tetramethylimidazoline-1-oxyl 3-oxide (PTIO), *S*-nitroso-*N*-acetyl-dl-penicillamine (SNAP), and *N*ω-nitro-l-arginine methyl ester hydrochloride (l-NAME).

The structures of the three ruthenium compounds, PTIO, SNAP, and l-NAME, are shown in Scheme 1.

### 4.2. Murine Peritoneal Macrophages

Murine peritoneal macrophages were obtained according to the procedure described by Zhang et al. [38]. To this purpose, adult male Centro di Biotecnologie Avanzate (CBA) mice from an established colony of the animal house of the University of Trieste were used. Animal studies were carried out according to the guidelines in force in Italy (DDL 116 of 21/2/1992 and subsequent addenda) and in compliance with the Guide for the Care and Use of Laboratory Animals, DHHS pub. No. (NIH) 86–23, Bethesda, MD: NIH, 1985. Briefly, donor mice were injected in the peritoneal cavity with 1 mL of a 3% thioglycollate solution in sterile water. After four days, mice were euthanized with CO_2_, the abdomen of each mouse soaked with 70% alcohol and a small incision carried out along the midline with sterile scissors, then the abdominal skin was retracted to expose the intact peritoneal wall. Five millilitres of cold PBS (Phosphate Buffered Saline pH = 7.4) were injected into the peritoneum of each mouse, and then the fluid was aspirated from the peritoneum and dispensed into a 50 mL centrifuge tube on ice. The peritoneal exudate cells were centrifuged at 400× *g* for 5 min at 4 °C, and the supernatant discarded and the cell pellet re-suspended in Roswell Park Memorial Insitute culture medium 1640 (RPMI) medium containing 0.25 M Hepes (EuroClone, Devon, UK) and 10^5^ cells/well seeded into a 96-well plate. After 2 h, the supernatant was discarded, cells were washed twice with PBS and macrophages maintained in RPMI culture medium supplemented with 10% foetal bovine serum (FBS, Invitrogen, Paisley, Scotland, UK), 2 mM l-glutamine (EuroClone), 100 IU/mL Penicillin, 100 μg/mL streptomycin (EuroClone), 0.5% gentamicin, 4 × 10^−4^ M sodium pyruvate (EuroClone), 0.25 M Hepes (EuroClone), and 1% non-essential amino acids (EuroClone).

### 4.3. EA.hy926 Cell Line

The human endothelial-like immortalised cell line EA.hy926, derived from the fusion of human umbelical vein endothelial cells (HUVEC) with the lung carcinoma cell line A549 was kindly provided by Mauro Coluccia, University of Bari, Bari, Italy, and was maintained in Dulbecco’s modified Eagle’s medium (DMEM, EuroClone), supplemented with 10% heat-inactivated FBS, 2 mM l-glutamine, 100 IU/mL penicillin and 100 μg/mL streptomycin.

The cell line was kept in an incubator with 5% CO_2_ and 100% relative humidity at 37 °C. Cells from a confluent monolayer were removed from flasks by a trypsin-EDTA solution. Cell viability was determined by the trypan blue dye exclusion test. For experimental purposes, cells were sown in flasks or in multi-well culture clusters.

### 4.4. Cytotoxicity Test

Cells were seeded at 5000 per well on 96-well plates and allowed to grow until they reached a sub-confluence stage. Then, they were incubated for 48 and 72 h with 10^−6^–10^−3^ M solutions of NAMI-A, RuEDTA, KP1339, PTIO, SNAP, and l-NAME obtained by serial dilution of a stock solution (freshly prepared in sterile water at a concentration of 2 mM) with complete medium containing 5% FBS. Analysis of cell cytotoxicity by MTT (3-(4,5-dimethylthiazol-2-yl)-2,5-diphenyltetrazolium bromide) assay was performed after 48 or 72 h of incubation. Briefly, MTT dissolved in PBS (5 mg/mL) was added (10 μL per 100 μL medium) to all wells, and the plates were then incubated at 37 °C with 5% CO_2_ and 100% relative humidity for 4 h. After this time, the medium was discarded and 200 μL of a 10% Igepal^®^ solution in HCl 0.01 N were added to each well for 30 min at 37 °C. Absorbance units were measured at λ = 570 nm on an Automated Microplate Reader EL311s (BIO-TEK^®^ Instruments, Winooski, VT, USA). IC_50_ values were calculated from dose–effect curves and are the mean ± standard deviation (S.D.) of at least three separate experiments. The fitting procedure applied is a nonlinear regression performed with GraphPad Prism version 6 for Mac OS X version 6.0b (GraphPad Software, San Diego, CA, USA). Experiments were conducted in quadruplicate and repeated three times.

### 4.5. Invasion Assay

Invasion assay was performed using 8.0 μm pore size Transwell^®^ inserts (Costar, Cambridge, MA, USA) coated with Matrigel™ (400 μg/mL, BD, Milano, Italy) at room temperature overnight. EA.hy926 cells were treated with NAMI-A, RuEDTA, SNAP, and l-NAME (10^−5^, 10^−4^, and 3 × 10^−4^ M) in serum-starved medium for 24 h at 37 °C, 5% CO_2_ before being seeded on inserts (10^5^ cells/insert) in the same medium containing 0.1% bovine serum albumine (BSA). As invasion stimulus, the complete medium was applied in the plate wells, as negative control to detect the basal invasion rate a serum-free medium was used. After 24 h, cells that had remained on the upper side of the membrane were removed using cotton swabs, while the cells that invaded and were present in the lower surface of the inserts were fixed with methanol, stained with May-Grünwald-Giemsa and observed at light microscopy (400×) (Orthoplan, Leitz, Wetzlar, Germany). Cells that have invaded have been counted in 7 fields. The results are expressed as percentage of treated/untreated cells.

### 4.6. Nitric Oxide Measurements

Murine peritoneal macrophages were seeded at 10^5^/well into 96-well plates in complete medium and treated with the compounds and with 10 μg/mL LPS (Lipopolysaccharide), accordingly to literature data reporting that, in the presence of LPS, the production of nitric oxide was found to be induced in macrophages in a time- and dose-dependent manner [16]. The NO production was measured by the Griess test.

EA.hy926 cells were seeded at 5000/well into 96-well plates in complete medium and treated with the compounds. The quantification of NO in the extracellular medium was measured by the Griess test while the fluorescent probe DAF-2 DA (Molecular Probes, Eugene, OR, USA) was used to detect NO at intracellular level.

### 4.7. Griess Test

Nitric oxide (NO) was determined on supernatants of cell cultures with Griess reagent according to Stuher and Nathan [39]. Briefly, supernatants of cultures (85 μL) were put in microtitre 96-well plates and added with 5 μL nitrate reductase and 10 μL NADH for 20 min at room temperature (RT), and then with 100 μL Griess reagent (1% naphtylethylendiamine to 1% sulphanilamide, 1:1). After 10 min at RT, the absorbance units were measured at 540 nm. NaNO_2_ was used as standard.

### 4.8. DAF-2 DA Fluorescent Probe

Diamminofluoresceins are usually used to detect NO in the intracellular milieu. DAF-2 DA has been widely applied to study NO in endothelial cells [40,41]. It enters by diffusion into cells where it is hydrolysed by cytosol esterases releasing the DAF-2 specie, which in the presence of NO and O_2_ is converted to the fluorescent derivative DAF-2T. The probe was added to the growth medium at 5 μM for 45 min at 37 °C and 5% CO_2_. The fluorescence (excitation at 492 nm and emission at 515 nm) was measured by a fluorimeter FluoroCount™ (Packard, Milano, Italy).

### 4.9. Matrigel™ Pellets Angiogenic Test

The experiment was carried out at the Centro di Biotecnologie Avanzate (CBA) of the University of Genua, in collaboration with the group of Adriana Albini. C57/BL 6 N male mice were implanted subcutaneously (s.c.) with 600 μL of Matrigel™ added with VEGF (36 ng/pellet), heparin (12 U/pellet), TNF-α (0.72 ng/pellet) and PBS (for the controls) or NAMI-A to obtain a final concentration of 2.4 × 10^−4^ M. Four days after the implant mice were sacrificed with CO_2_, and the pellets extracted, photographed and processed for the determination of the haemoglobin content. Briefly, the pellets were put in eppendorf tubes with water and disaggregated mechanically with the aid of scissors. After centrifugation the supernatant was collected and the haemoglobin content determined with a kit purchased from Sigma and based on the method of Drabkin [42]. The procedure is a colorimetric cyanomethaemoglobin method where total haemoglobin at alkaline pH is rapidly converted to the cyanoderivative. The absorbance of the cyanoderivative is determined at 540 nm. The haemoglobin content was then normalized to the weight of the pellets.

### 4.10. Statistical Analysis

Results obtained were processed using InstatGraph3 software (Version 3.0, GraphPad Software Inc., San diego, CA, USA) and presented as mean ± mean ± standard error medium (S.E.M.) The group means were compared using a Two-Way Analysis of Variance (ANOVA) followed by Tukey–Kramer post-test and considered significant when *p* < 0.05.

## Figures and Tables

**Table 1:** IC_50_ values of imidazolium *trans*-imidazoledimethylsulfoxidetetrachloro-ruthenate (NAMI-A), potassiumchlorido(ethylendiamminotetraacetate)rutenate(III) (RuEDTA), sodium (bis-indazole)tetrachloro-ruthenate(III), Na[*trans*-RuCl_4_Ind_2_] (KP1339), 2-phenyl-4,4,5,5-tetramethylimidazoline-1-oxyl 3-oxide (PTIO), *S*-nitroso-*N*-acetyl-dl-penicillamine (SNAP), and *N*ω-nitro-l-arginine methyl ester hydrochloride (l-NAME) in EA.hy926 cells.

Compound	IC_50_ [μM]	
	48 h	72 h
NAMI-A	960 (623–1481)	360 (150–885)
RuEDTA	>1000	880 (507–1534)
KP1339	37 (20–68)	22 (12–38)
PTIO	87	94
SNAP	500 (85–2893)	220 (85–599)
l-NAME	>1000	>1000

EA.hy926 cells were treated with NAMI-A, RuEDTA, KP1339, PTIO, SNAP, and l-NAME at 10^−6^–10^−3^ M for 48 or 72 h before measuring cell viability by 3-(4,5-dimethylthiazol-2-yl)-2,5-diphenyltetrazolium bromide (MTT) test. Values in parentheses represent the 95% Confidence Intervals.

## References

[1] Hanahan D., Folkman J. (1996). Patterns and emerging mechanisms of the angiogenic switch during tumorigenesis. Cell.

[2] Ferrara N. (2009). Vascular endothelial growth factor. Arterioscler. Thromb. Vasc. Biol..

[3] Giacomini A., Chiodelli P., Matarazzo S., Rusnati M., Presta M. (2016). Blocking the FGF/FGFR system as a “two-compartment” antiangiogenic/antitumor approach in cancer therapy. Pharmacol. Res..

[4] Zhao Y., Adjei A.A. (2015). Targeting angiogenesis in cancer therapy: Moving beyond vascular endothelial growth factor. Oncologist.

[5] Keating G.M. (2014). Bevacizumab: A review of its use in advanced cancer. Drugs.

[6] Rock E.P., Goodman V., Jiang J.X., Mahjoob K., Verbois S.L., Morse D., Dagher R., Justice R., Pazdur R. (2007). Food and Drug Administration drug approval summary: Sunitinib malate for the treatment of gastrointestinal stromal tumor and advanced renal cell carcinoma. Oncologist.

[7] Sherbet G.V. (2015). Therapeutic potential of thalidomide and its analogues in the treatment of cancer. Anticancer Res..

[8] Vacca A., Bruno M., Boccarelli A., Coluccia M., Ribatti D., Bergamo A., Garbisa S., Sartor L., Sava G. (2002). Inhibition of endothelial cell functions and of angiogenesis by the metastasis inhibitor NAMI-A. Br. J. Cancer.

[9] Morbidelli L., Donnini S., Filippi S., Messori L., Piccioli F., Orioli P., Sava G., Ziche M. (2003). Anti angiogenic properties of selected ruthenium(III) complexes that are nitric oxide scavengers. Br. J. Cancer.

[10] Sanna B., Dedidda M., Pintus G., Tadolini B., Posadino A.M., Bennardini F., Sava G., Ventura C. (2002). The anti-metastatic agent imidazolium *trans-*imidazoledimethylsulfoxide-tetrachlororuthenate induces endothelial cell apoptosis by inhibiting the mitogen-activated protein kinase/extracellular signal-regulated kinase signalling pathway. Arch. Biochem. Biophys..

[11] Pintus G., Tadolini B., Posadino A.M., Sanna B., Debidda M., Bennardini F., Sava G., Ventura C. (2002). Inhibition of the MEK/ERK signalling pathway by the novel antimetastatic agent NAMI-A down regulates *c-myc* gene expression and endothelial cell proliferation. Eur. J. Biochem..

[12] Cocchietto M., Sava G. (2000). Blood concentration and toxicity of the antimetastasis agent NAMI-A following repeated intravenous treatment in mice. Pharmacol. Toxicol..

[13] Ziche M., Morbidelli L., Masini B., Amerini S., Granger H.J., Maggi C.A., Geppetti P., Ledda F. (1994). Nitric oxide mediates angiogenesis in vivo and endothelial cell growth and migration in vitro promoted by substance P. J. Clin. Investig..

[14] Ziche M., Morbidelli L., Choudhuri R., Zhang H.T., Donnini S., Granger H.J., Bicknell R. (1997). Nitric oxide synthase lies downstream from vascular endothelial growth factor-induced but not basic fibroblast growth factor-induced angiogenesis. J. Clin. Investig..

[15] Parenti A., Morbidelli L., Cui X.L., Douglas J.G., Hood J., Granger H.J., Ledda F., Ziche M. (1998). Nitric oxide is an upstream signal for vascular endothelial growth factor-induced extracellular signal-regulated kinases^1^/_2_ activation in postcapillary endothelium. J. Biol. Chem..

[16] Krol W., Czuba Z.P., Threadgill M.D., Cunningham B.D.M., Pietsz G. (1995). Inhibition of nitric oxide (NO·) production in murine macrophages by flavones. Biochem. Parmacol..

[17] Edgell C.J., McDonald C.C., Graham J.B. (1983). Permanent cell line expressing human factor VIII-related antigen established by hybridisation. Proc. Natl. Acad. Sci. USA.

[18] Keppler B.K., Henn M., Juhl U.M., Berger M.R., Niebl R., Wagner F.E. (1989). New ruthenium complexes for the treatment of cancer. Progress in Clinical Biochemistry and Medicine.

[19] Sava G., Pacor S., Zorzet S., Alessio E., Mestroni G. (1989). Antitumour properties of dimethylsulphoxide ruthenium(II) complexes in the Lewis Lung Carcinoma system. Pharmacol. Res..

[20] Sava G., Pacor S., Mestroni G., Alessio E. (1992). Effects of the Ru(III) complexes [mer-RuCl_3_(DMSO)2Im][degrees] and Na[*trans*-RuCl4(DMSO)Im] on solid mouse tumors. Anti-Cancer Drugs.

[21] Sava G., Pacor S., Bergamo A., Cocchietto M., Mestroni G., Alessio E. (1995). Effects of ruthenium complexes on experimental tumours: Irrelevance of cytotoxicity for metastasis inhibition. Chem. Biol. Interact..

[22] Sava G., Clerici K., Capozzi I., Cocchietto M., Gagliardi R., Alessio E., Mestroni G., Perbellini A. (1999). Reduction of lung metastasis by ImH[*trans*-RuCl_4_(DMSO)Im]: Mechanism of the selective action investigated on mouse tumors. Anti-Cancer Drugs.

[23] Sava G., Capozzi I., Clerici K., Gagliardi R., Alessio E., Mestroni G. (1998). Pharmacological control of lung metastases of solid tumours by a novel ruthenium complex. Clin. Exp. Metastasis.

[24] Sava G., Zorzet S., Turrin C., Vita F., Soranzo M.R., Zabucchi G., Cocchietto M., Bergamo A., DiGiovine S., Pezzoni G. (2003). Dual action of NAMI-A in inhibition of solid tumor metastasis: Selective targeting of metastatic cells and binding to collagen. Clin. Cancer Res..

[25] Coluccia M., Sava G., Salerno G., Bergamo A., Pacor S., Mestroni G., Alessio E. (1995). Efficacy of 5-FU combined to Na[*trans*-RuCl_4_(DMSO)Im], a novel selective antimetastatic agent, on the survival time of mice with p388 leukemia, P388/DDP subline and MCa mammary carcinoma. Metal-Based Drugs.

[26] Zorzet S., Bergamo A., Cocchietto M., Sorc A., Gava B., Alessio E., Iengo E., Sava G. (2000). Lack of in vitro cytotoxicity, associated to increased G_2_-M cell fraction and inhibition of matrigel invasion, may predict in vivo-selective antimetastasis activity of ruthenium complexes. J. Pharmacol. Exp. Ther..

[27] Gava B., Zorzet S., Spessotto P., Cocchietto M., Sava G. (2006). Inhibition of B16 melanoma metastases with the ruthenium complex imidazolium *trans*-imidazoledimethylsulfoxide-tetrachlororuthenate and down-regulation of tumor cell invasion. J. Pharmacol. Exp. Ther..

[28] Goligorsky M.S., Budzikowski A.S., Tsukahara H., Noiri E. (1999). Co-operation between endothelin and nitric oxide in promoting endothelial cell migration and angiogenesis. Clin. Exp. Pharmacol. Physiol..

[29] Franke A., Oszajca M., Brindell M., Stochel G., van Eldik R. (2015). Metal-assisted activation of nitric oxide-mechanistic aspecte of complex nitrosylation processes. Adv. Inorg. Chem..

[30] Oszajca M., Kulis E., Stochel G., Brindell M. (2014). Interaction of the NAMI-A complex with nitric oxide under physiological conditions. New J. Chem..

[31] Bucinsky L., Büchel G.E., Ponec R., Rapta P., Breza M., Kozisek J., Gall M., Biskupic S., Fronc M., Schiessl K. (2013). On the electronic structure of *mer*,*trans*-[RuCl_3_(1*H*-indazole)_2_(NO)], a hypothetical metabolite of the antitumor drug candidate KP1019: An experimental and DFT study. Eur. J. Inorg. Chem..

[32] Das D., Mondal P. (2015). Quantum chemical studies on detail mechanism of nitrosylation of NAMI-A-HSA adduct. J. Phys. Chem..

[33] Aitken J.B., Antony S., Weekley C.M., Lai B., Spiccia L., Harris H.H. (2012). Distinct cellular fates for KP1019 and NAMI-A determined by X-ray fluorescence imaging of single cells. Metallomics.

[34] Rademaker-Lakhai J.M., van den Bongard D., Pluim D., Beijnen J.H., Schellens J.H. (2004). A Phase I and pharmacological study with imidazolium-*trans*-DMSO-imidazole-tetrachlororuthenate, a novel ruthenium anticancer agent. Clin. Cancer Res..

[35] Bergamo A., Gerdol M., Lucafò M., Pelillo C., Battaglia M., Pallavicini A., Sava G. (2015). RNA-seq analysis of the whole transcriptome of MDA-MB-231 mammary carcinoma cells exposed to the antimetastatic drug NAMI-A. Metallomics.

[36] Mestroni G., Alessio E., Sava G. (1998). New Salt of Anionic Complexes of Ru(III) as Antimetastatic and Antineoplastic Agents. International Patent.

[37] Diamantis A.A., Dubrawsky J.V. (1981). Preparation and structure of ethylenediaminetetraacetate complexes of ruthenium(II) with dinitrogen, carbon monoxide, and other π-acceptor ligands. Inorg. Chem..

[38] Zhang X., Goncalves R., Mosser D.M. (2008). The isolation and characterization of murine macrophages. Curr. Protoc. Immunol..

[39] Stuehr D.J., Nathan C.F. (1989). Nitric oxide. A macrophage product responsible for cytostasis and respiratory inhibition in tumor target cells. J. Exp. Med..

[40] Itoh Y., Ma F.H., Hoshi H., Oka M., Noda K., Ukai Y., Kojima H., Nagano T., Toda N. (2000). Determination and bioimaging method for nitric oxide in biological specimens by diaminofluorescein fluorimetry. Anal. Biochem..

[41] Nakatsubo N., Kojima H., Kikuchi K., Nagoshi H., Hirata Y., Maeda D., Imai Y., Irimura T., Nagano T. (1998). Direct evidence of nitric oxide production from bovine aortic endothelial cells using new fluorescence indicators: Diaminofluoresceins. FEBS Lett..

[42] Drabkin D.L., Austin J.H. (1935). Spectrophotometric studies II. Preparations from washed blood cells; nitric oxide haemoglobin and sulfhemoglobin. J. Biol. Chem..

